# Review of metastatic colorectal cancer treatment pathways and early clinical experience of trifluridine/tipiracil in the UK named patient programme

**DOI:** 10.1186/s12885-020-6577-1

**Published:** 2020-02-03

**Authors:** Timothy Iveson, Angela M. Carter, Kai-Keen Shiu, Clare Spooner, Daniel Stevens, Saifee Mullamitha

**Affiliations:** 1 University Hospital Southampton NHS Foundation Trust, Southampton, UK; 2 OPEN VIE, Marlow, UK; 3 University College Hospitals NHS Foundation Trust, London, UK; 4 Medical Affairs, Servier Laboratories UK, Stoke Poges, Slough, UK; 5 The Christie NHS Foundation Trust, Manchester, UK

**Keywords:** metastatic colorectal cancer, treatment pathways, real-world data, trifluridine and tipiracil, named patient programme, disease progression, treatment duration

## Abstract

**Background:**

The standard first- and second- line chemotherapy backbone regimens for metastatic colorectal cancer (mCRC) are 5-fluorouracil (5-FU)/capecitabine-based with addition of irinotecan or oxaliplatin. Until recently, evidence for optimal sequencing post second-line was sparse. Trifluridine/tipiracil (indicated for mCRC and gastric cancer after standard chemotherapies) was made available to UK patients via a named patient programme (NPP) before receiving marketing authorisation in Europe in 2016, allowing characterisation of UK treatment pathways, and evaluation of trifluridine/tipiracil in a UK non-trial population.

**Methods:**

Data collected routinely for the NPP were analysed to describe the patient demographics, clinical characteristics and treatment pathways. Patients eligible for the programme were adults (≥18 years) with histologically or cytologically confirmed mCRC who had previously received chemotherapy treatment(s).

**Results:**

Of the 250 eligible patients enrolled in the NPP, 194 patients received ≥1 dose of trifluridine/tipiracil and 56 patients did not receive trifluridine/tipiracil. The following results are reported first for patients who received trifluridine/tipiracil and second for those who did not receive trifluridine/tipiracil: median (IQR) age was 63.0 (54.0–69.0) and 62.0 (54.8–69.0) years; Eastern Cooperative Oncology Group performance status score was 0 for 28 and 14%, 1 for 65 and 70%, 2 for 7 and 16%. In terms of previous systemic treatments 47 and 43% had 2 prior lines of therapy. FOLFOX-, FOLFIRI- and CAPOX-based therapies were the most common first-line regimens in patients receiving trifluridine/tipiracil (37, 35 and 21%, respectively), and in patients not receiving trifluridine/tipiracil (41, 30 and 20%, respectively). Second-line treatment regimens in patients receiving and not receiving trifluridine/tipiracil were most commonly FOLFIRI-based (48 and 41%, respectively) and FOLFOX-based (19 and 21%, respectively). Patients received a median of 2 cycles of trifluridine/tipiracil with a median treatment duration of 1.8 (95% CI: 1.8–2.4) months. In patients who discontinued treatment due to disease progression, the median progression-free duration was 2.8 (95% CI: 2.4–2.9) months.

**Conclusions:**

The results highlight the number of treatment pathways used to treat mCRC in routine UK clinical practice prior to the marketing authorisation and National Institute for Health and Care Excellence approval of trifluridine/tipiracil and highlight the lack of clinical guidelines for mCRC.

## Background

Colorectal cancer is the fourth most common cancer diagnosed in the United Kingdom (UK) [1], accounting for approximately 12% of new cancer cases per year and 16,384 deaths in 2015, with the highest incidence in patients aged ≥85 years [2]. Metastatic colorectal cancer (mCRC) has a poor prognosis, with 5-year survival rates of approximately 14% [3]. The main first- and second-line chemotherapy regimens for mCRC recommended by the National Institute for Health and Care Excellence (NICE) are 5-fluorouracil (5-FU)- /capecitabine- based combination therapies, including: FOLFOX (5-FU, oxaliplatin and folinic acid), FOLFIRI (5-FU, folinic acid and irinotecan) and CAPOX (capecitabine and oxaliplatin) [4]. Targeted biologic treatments, such as the anti-epidermal growth factor receptor (EGFR) treatments, cetuximab and panitumumab, are also recommended for first-line treatment of RAS wild-type mCRC [5]. A range of potential post- second-line agents exist, including EGFR inhibitors (cetuximab/panitumumab), bevacizumab, regorafenib and trifluridine/tipiracil [6, 7]; however, EGFR inhibitors are not currently recommended by NICE beyond first line, and therefore are not reimbursed in the UK [8], whilst regorafenib and bevacizumab are not currently available for use in mCRC within the UK irrespective of treatment line.

Trifluridine/tipiracil is an orally administered 2:1 combination of trifluridine, an antineoplastic nucleoside analogue that is incorporated in DNA after phosphorylation and induces DNA dysfunction, and tipiracil hydrochloride, a thymidine phosphorylase inhibitor that increases the bioavailability of trifluridine [9]. The phase 3 RECOURSE clinical trial of trifluridine/tipiracil in patients with mCRC who had received at least 2 prior chemotherapy regimens (including fluoropyrimidine, irinotecan, oxaliplatin and bevacizumab) showed prolonged survival in patients treated with trifluridine/tipiracil (median overall survival [OS] of 7.1 months) compared with placebo (median OS of 5.3 months) [10]. The most commonly reported adverse event (AE) of grade 3 or higher in patients treated with trifluridine/tipiracil was neutropenia (38%), which was not observed in the placebo-treated group. Subsequent post-hoc analysis of RECOURSE trial data has demonstrated the clinical efficacy and tolerability of trifluridine/tipiracil in various subgroups [11] and clinically meaningful improvements in quality-adjusted survival [12]. There is further evidence supporting the clinical effectiveness and tolerability of trifluridine/tipiracil in various geographies [13–18].

Trifluridine/tipiracil received marketing authorisation for use in mCRC from the European Medicines Agency (EMA) in April 2016, and more recently for the treatment of patients with metastatic gastric cancer [19]. Trifluridine/tipiracil was subsequently found to be cost-effective in the UK by NICE in August 2016 for patients with mCRC who have been previously treated with, or are not considered candidates for, available therapies including fluoropyrimidine-, oxaliplatin- and irinotecan-based chemotherapies, anti-vascular endothelial growth factor (VEGF) agents, and anti-EGFR agents [19, 20]. For a period of 8 months from November 2015 trifluridine/tipiracil was available to UK patients with mCRC via a named patient programme (NPP), under Medicines and Healthcare products Regulatory Agency (MHRA) Guidance for Supply of Unlicensed Medicines (“specials”) [21].

There is a lack of data regarding the use of trifluridine/tipiracil in a non-trial UK population and information about the treatments being used in contemporary UK practice is scarce [22]. The purpose of the present analysis was to describe the characteristics and treatment pathways of patients with mCRC enrolled in this NPP in the UK.

## Methods

The NPP for trifluridine/tipiracil was available throughout the UK and Channel Islands prior to marketing authorisation; 51 centres (both National Health Service [NHS] and private) took part (42 in England, 3 in Wales, 5 in Scotland and 1 in the Bailiwick of Jersey). Patients were enrolled between 18th November 2015 and 15th July 2016.

The main objective of the present analysis was to describe the clinical characteristics, treatment pathways and trifluridine/tipiracil treatment of patients with mCRC treated with trifluridine/tipiracil in routine clinical practice in the UK. An additional objective was to describe the clinical characteristics and treatment pathways of patients who were enrolled in the NPP but did not subsequently receive trifluridine/tipiracil.

### Patients

Patients eligible for inclusion in the NPP were adults (≥ 18 years) with histologically or cytologically confirmed mCRC who had been previously treated with, or were not deemed candidates for, available therapies including 5-fluorouracil (5-FU)/capecitabine- based combination therapies, anti-VEGF agents, and anti-EGFR agents. Female patients must have had a negative pregnancy test within 7 days of trifluridine/tipiracil initiation (or been post-menopausal) and all patients were required to use adequate contraception (where possible), during and up to 6 months after treatment. Patients with severe renal impairment, moderate or severe hepatic impairment and those with reduced neutrophil counts or unresolved prior grade 3 or 4 non-haematological clinically relevant toxicity from prior therapies were excluded, consistent with the United States Prescribing Information (USPI, provided in lieu of the summary of product characteristics [SmPC] [19]).

Eligible patients were proposed by their treating physician and provided written informed consent to participate in the NPP; data were collected as part of the routine management of the NPP using paper data collection forms. The data collected included patient demographics and clinical characteristics (including age, sex, disease duration, *KRAS* mutation status, Eastern Cooperative Oncology Group Performance Status [ECOG-PS] score at enrolment), prior CRC treatment and trifluridine/tipiracil treatment (where appropriate; including number of cycles of trifluridine/tipiracil provided, reason for discontinuation, AEs). The recommended starting dose of trifluridine/tipiracil was 35 mg/m^2^ orally twice daily on Days 1 to 5 and on Days 8 to 12 in 28-day cycles, as indicated in the USPI/SmPC [19].

### Statistical analyses

Data were collected routinely as part of the NPP and no formal sample size power calculation was carried out; all patients enrolled in the NPP were included in the present analysis. Quantitative variables are presented as mean (standard deviation [SD]) or median (inter-quartile range [IQR] or range); categorical variables are presented as frequencies and percentages. Treatment persistence was defined as the time from initiation of trifluridine/tipiracil to the date of last intake or, where date of last intake was not recorded, duration was estimated from the number of cycles (28 days per cycle) of trifluridine/tipiracil received; patients continuing on treatment or transferring to funded treatment were censored on the last day of the last cycle received as part of the NPP. Treatment persistence is presented as a Kaplan–Meier plot with median (95% confidence intervals [95% CI]). For patients who discontinued trifluridine/tipiracil due to disease progression, in the subgroup of patients with an available date of scan confirming disease progression (*n* = 58), the time from initiation to disease progression was highly correlated with treatment persistence (*r* = 0.984). Therefore, time to progression was defined as the time from initiation of trifluridine/tipiracil to the date of disease progression, or where date of disease progression was not recorded, treatment persistence was used as a proxy for time to progression; patients not discontinuing due to disease progression were censored on the last day of the last cycle received as part of the NPP. Progression-free status is presented as median (95% CI). For the purposes of treatment sequencing, treatments were grouped based on the primary treatment (e.g. FOLFIRI includes FOLFIRI only and FOLFIRI with additional agents).

## Results

### Study population

A total of 250 patients enrolled in the NPP were included in the present analyses; of these, 194 received at least one dose of trifluridine/tipiracil and 56 patients were enrolled but did not receive trifluridine/tipiracil because they subsequently declined treatment or they rapidly became too unwell from their progressing disease to receive treatment. The patient demographics and clinical characteristics of patients receiving at least one dose of trifluridine/tipiracil and those not receiving this treatment are summarised in Table 1.

**Table 1:** Patient demographics and clinical characteristics at enrolment into the UK named patient programme

	Patients receiving ≥1 dose of trifluridine/tipiracil (*n* = 194)	Patients not commencing treatment (*n* = 56)
Age (years), median (IQR)	63.0 (54.0–69.0)	62.0 (54.8–69.0)
Female, *n* (%)	83 (43%)	21 (38%)
BMI (kg/m^2^), median (IQR)	25.8 (22.7–29.6)	26.0 (23.5–28.4)
ECOG-PS, *n* (%)		
0	54 (28%)	8 (14%)
1	127 (65%)	39 (70%)
2	13 (7%)	9 (16%)
KRAS-mutation, *n* (%)		
Wild type	89 (46%)	25 (45%)
Mutated	74 (38%)	25 (45%)
Not known	17 (9%)	4 (7%)
Not recorded	14 (7%)	2 (4%)
Disease duration (months),^a^ median (IQR)	35.7 (18.7–54.7)	33.4 (19.5–47.9)
Time from diagnosis of metastatic disease		
< 18 months	61 (31%)	20 (36%)
≥ 18 months	133 (69%)	36 (64%)
Prior colorectal surgery (n, %)		
Yes	143 (74%)	36 (64%)
No	51 (26%)	20 (36%)
Neoadjuvant therapy (n, %)		
Yes	4 (2%)	1 (2%)
No	190 (98%)	55 (98%)
Adjuvant therapy (n, %)		
Yes	75 (39%)	16 (29%)
No	119 (61%)	40 (71%)
Number of prior lines of therapy for metastatic disease		
1	15 (8%)	6 (11%)
2	92 (47%)	24 (43%)
3	51 (26%)	12 (21%)
≥ 4	36 (19%)	14 (25%)
Prior therapies for metastatic disease		
Fluorouracil or capecitabine	193 (99%)	55 (98%)
*5-FU*	*178 (92%)*	*53 (95%)*
*Capecitabine*	*90 (46%)*	*26 (46%)*
Oxaliplatin	163 (84%)	49 (88%)
Irinotecan	193 (99%)	55 (98%)
Bevacizumab	88 (45%)	20 (36%)
Cetuximab	65 (34%)	17 (30%)
Panitumumab	10 (5%)	2 (4%)
Aflibercept	30 (15%)	9 (16%)
Regorafenib	9 (5%)	1 (2%)
Mitomycin or MMC	21 (11%)	4 (7%)
Other	26 (13%)	9 (16%)

^a^Time between date of initial CRC diagnosis and enrolment

In patients receiving ≥1 dose of trifluridine/tipiracil, the median age at enrolment was 63.0 (IQR 54.0–69.0) years (43% female, *n* = 83/194) with a median disease duration (defined as the time from initial diagnosis of colorectal cancer to enrolment) of 35.7 (IQR 18.7–54.7) months; the median time from diagnosis of metastatic disease to enrolment was 25.4 (IQR 16.4–43.0) months. Twenty eight percent (*n* = 54/194) had an ECOG-PS score of 0, and 74% had prior colorectal surgery (*n* = 143/194); 39% (*n* = 75/194) of patients had received adjuvant therapy and 2% (*n* = 4/194) had received neoadjuvant therapy. Patients enrolled but not receiving trifluridine/tipiracil had broadly similar characteristics, with a median age of 62.0 (IQR 54.8–69.0) years (38% female, *n* = 21/56), a median disease duration of 33.4 (IQR 19.5–47.9) months and a median time from diagnosis of metastatic disease to enrolment of 22.4 (IQR 15.6–43.0) months. Of the patients enrolled but not receiving trifluridine/tipiracil, 14% (*n* = 8/56) had an ECOG-PS score of 0, and 64% (*n* = 36/56) had prior colorectal surgery; 29% (*n* = 16/56) of patients had received adjuvant therapy and 2% (*n* = 1/56) had received neoadjuvant therapy.

### Treatment pathways

Of the patients receiving ≥1 dose of trifluridine/tipiracil, 19% (*n* = 36/194) had received at least 4 prior therapies (Table 1); in patients who did not receive trifluridine/tipiracil, 25% (*n* = 14/56) had received at least 4 prior therapies.

**Fig. 1:**
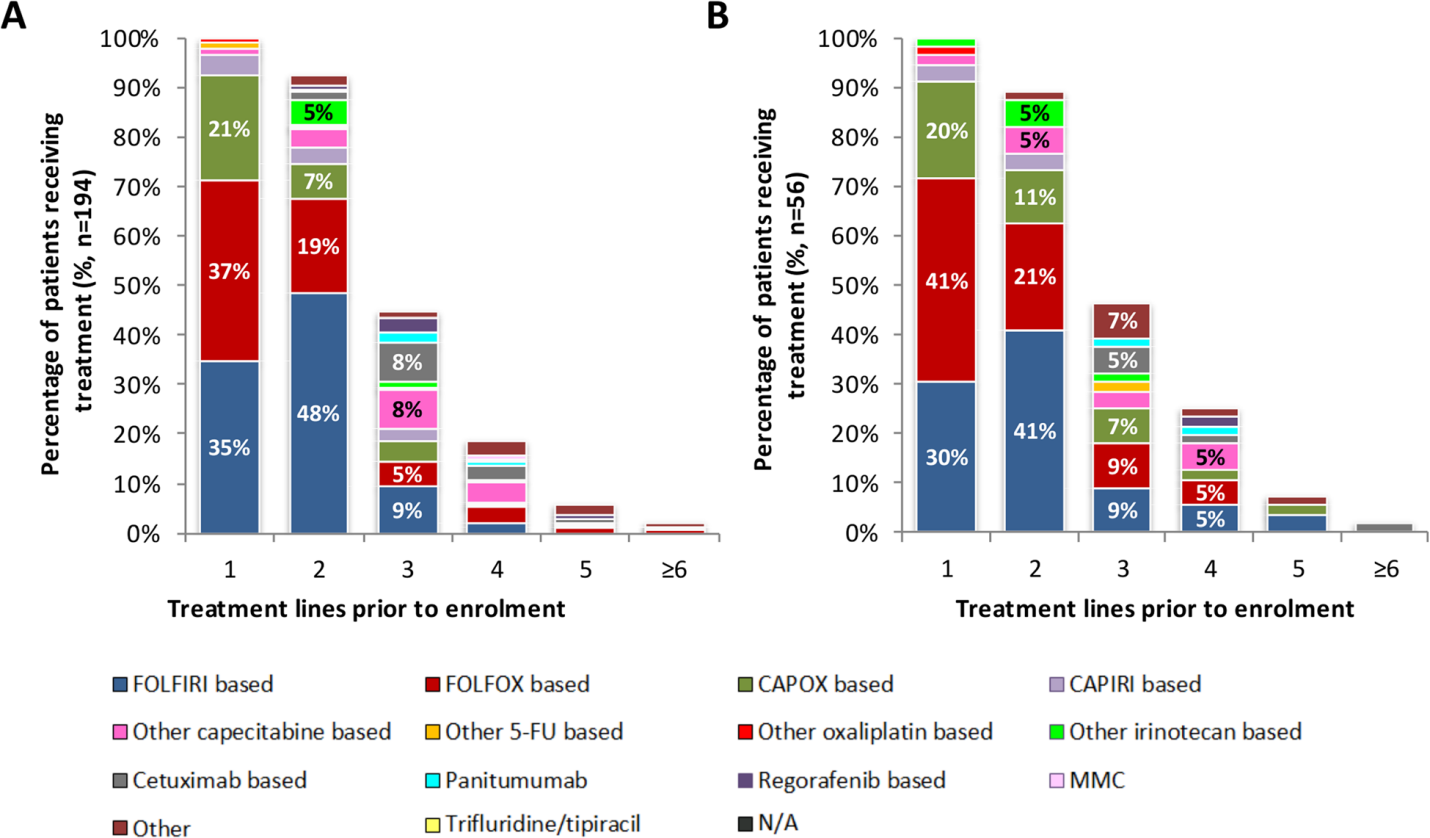
Prior treatment lines in patients with mCRC enrolled in the UK named patient programme. **a** Prior treatment lines for mCRC before trifluridine/tipiracil in patients receiving ≥1 dose; **b** Prior treatment lines for mCRC in patients who did not receive trifluridine/tipiracil. Percentages are not presented for groups with < 5% of patients. Treatments were grouped based on primary treatment. Treatment regimens were grouped as follows: (i) ‘FOLFIRI based’ includes FOLFIRI only or in combination with one or more of the following: aflibercept, bevacizumab, capecitabine, cetuximab, panitumumab. (ii) ‘FOLFOX based’ includes FOLFOX only or in combination with one or more of the following: bevacizumab, capecitabine, cetuximab, irinotecan, other. (iii) ‘CAPOX based’ includes CAPOX only or CAPOX with bevacizumab and/or other. (iv) ‘CAPIRI based’ includes CAPIRI only or CAPIRI with bevacizumab or cetuximab. (v) ‘Other capecitabine based’ includes capecitabine only or in combination with one or more of the following: aflibercept, bevacizumab, mitomycin, other. (vi) ‘Other 5-FU based’ includes 5-fluorouracil (5-FU) only or 5-FU plus capecitabine or other. (vii) ‘Other oxaliplatin based’ includes oxaliplatin only, oxaliplatin with irinotecan and cetuximab and oxaliplatin with other. (viii) ‘Other irinotecan based ‘ includes irinotecan only or in combination with one or more of the following: aflibercept, bevacizumab, cetuximab, other. (ix) ‘Cetuximab based’ includes cetuximab only or in combination with panitumumab. (x) ‘Regorafenib based’ includes regorafenib only or with other

The treatment regimens prior to enrolment in the NPP are summarised in Fig. 1. A total of 26 different first-line treatment regimens were recorded (see Additional file 1: Table S1). FOLFOX-, FOLFIRI- and CAPOX-based therapies were the most common first-line regimens in patients receiving ≥1 dose of trifluridine/tipiracil (37% [*n* = 71/194], 35% [*n* = 67/194] and 21% [*n* = 41/194], respectively (Fig. 1a)), and in patients not receiving trifluridine/tipiracil (41% [*n* = 23/56], 30% [*n* = 17/56] and 20% [*n* = 11/56], respectively (Fig. 1b)). Second-line treatment regimens in patients receiving and not receiving ≥1 dose of trifluridine/tipiracil were most commonly FOLFIRI-based (48% [*n* = 94/194] and 41% [*n* = 23/56], respectively) and FOLFOX-based (19% [*n* = 37/194] and 21% [*n* = 12/56], respectively). Treatment beyond the second-line was highly varied, with a wide range of different treatment regimens observed (Fig. 1). The most common third-line treatments in patients receiving ≥1 dose of trifluridine/tipiracil were FOLFIRI-, capecitabine- or cetuximab-based regimens (9% [*n* = 18/194], 8% [*n* = 15/194] and 8% [*n* = 16/194], respectively). Patients not receiving trifluridine/tipiracil most commonly received FOLFIRI- or FOLFOX-based regimens (9% [*n* = 5/56] each) at third line.

First- and second-line treatment sequencing is shown in Additional file 1: Figure S1. For those patients receiving trifluridine/tipiracil, 17% (*n* = 33/194) of patients had FOLFIRI-based first-line followed by FOLFOX-based second-line regimens, 30% (*n* = 58/194) had FOLFOX-based first-line followed by FOLFIRI-based second-line regimens and 13% (*n* = 26/194) had CAPOX-based first-line followed by FOLFIRI-based second-line regimens (Additional file 1: Figure S1A). Seven percent (n = 14/194) had FOLFIRI-based first-line regimens followed by trifluridine/tipiracil as second-line regimen. For those patients not receiving trifluridine/tipiracil, 14% (n = 8/56) of patients had FOLFIRI-based first-line followed by FOLFOX-based second-line regimens, 23% (n = 13/56) had FOLFOX-based first-line followed by FOLFIRI-based second-line regimens and 16% (n = 9/56) had CAPOX-based first-line followed by FOLFIRI-based second-line regimens (Additional file 1: Figure S1B). All other combinations of first- and second-line regimen were observed in < 5% of patients in each group.

### Treatment persistence

**Fig. 2:**
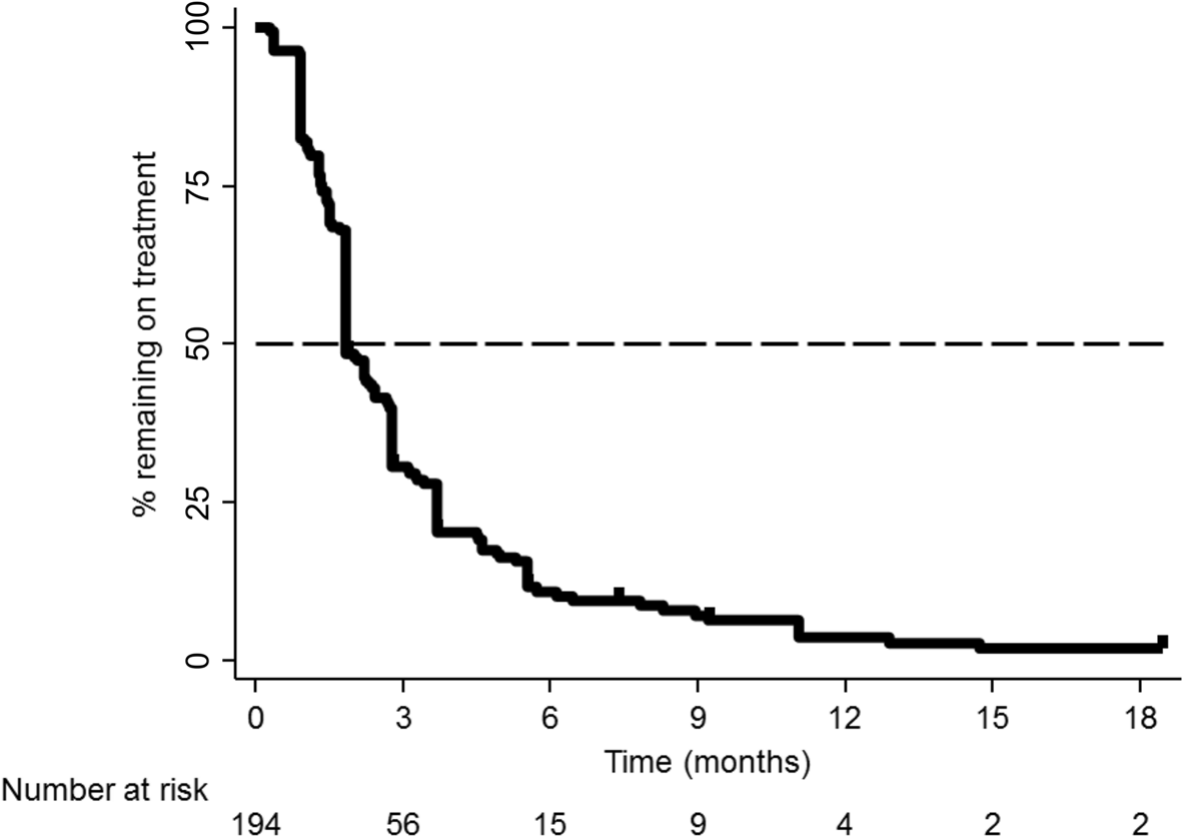
Trifluridine/tipiracil treatment persistence

Patients received a median of 2 cycles of trifluridine/tipiracil (range 1–20). The median treatment persistence was 1.8 (95% CI: 1.8–2.4) months (Fig. 2), with 30% (95% CI 24–37%) of patients persisting on treatment at 3 months, 10% (95% CI: 6–15%) at 6 months and 3% (95% CI: 1–8%) at 12 months. Overall, 92% (*n* = 179/194) of patients discontinued trifluridine/tipiracil, most commonly due to disease progression (79% [*n* = 142/179]); 3% (*n* = 5/179) discontinued due to AEs and 2% (*n* = 4/179) due to other reasons (16% [*n* = 28/179] reason for discontinuation unknown). Of the 15 patients remaining on trifluridine/tipiracil treatment at data collection, 87% (*n* = 13/15) had switched to commercial stock. In patients who discontinued treatment due to disease progression, the median progression-free duration was 2.8 (95% CI: 2.4–2.9) months.

### Adverse events

Of the patients receiving ≥1 dose of trifluridine/tipiracil, 55% (*n* = 106/194) experienced at least one adverse event. Neutropenia (all grades) was the most commonly observed AE, reported in 30% (*n* = 59/194) of patients (Table 2).

**Table 2:** Adverse events

Adverse events (patients receiving ≥ 1 dose of trifluridine/tipiracil)	*n*	% (*n* = 194)
≥1 AE	106	55%
*Deranged renal/creatinine*	4	2%
*Anaemia*	12	6%
*Neutropenia*	59	30%
*Neutropenic sepsis*	8	4%
*Thrombocytopenia*	8	4%
*Pancytopenia*	2	1%
*Leukopenia*	10	5%
*Lymphocytopenia/lymphopenia*	2	1%
*Hyperbilirubinaemia*	3	2%
*Other*	71	37%

## Discussion

The results of this analysis of data from the trifluridine/tipiracil NPP demonstrate the complex treatment patterns of patients with mCRC. The demographics and clinical characteristics of patients enrolled in the NPP who went on to receive at least one dose of trifluridine/tipiracil were broadly similar to those not receiving trifluridine/tipiracil. Furthermore, patients enrolled had similar demographic characteristics (median age, sex) to those in the RECOURSE trial [10] and several real-world studies [13–16]. However, there was a higher proportion of patients in the RECOURSE trial [10] and the Italian single-centre compassionate use programme (CUP) [13] with ECOG-PS scores of 0 (56 and 63% respectively) than patients enrolled in the UK NPP (28% of patients receiving ≥1 dose of trifluridine/tipiracil, 14% of patients not receiving trifluridine/tipiracil). The patients enrolled in the UK NPP were more similar to the sample reported in the German CUP, in which 28% of patients had ECOG scores of 0 [17]. The reasons for these differences are unclear but may reflect differing clinical practice in the evaluation and recording of ECOG-PS scores in different countries, or may suggest that patients included in the German and UK NPPs were at a more advanced stage of disease at enrolment.

The number and type of previous treatments observed were similar to those reported in observational studies from the Netherlands [14], Japan [15], Latvia [16] and Germany [17]. However, a smaller proportion of the patients receiving trifluridine/tipiracil in this analysis had at least 4 prior treatment regimens (19%) than in the RECOURSE trial [10] (60%). We observed a wide range of treatment regimens at all lines of therapy. Similar to the populations reported in Skuja et al. [16] and the RECOURSE trial [10], most (≥90%) patients had previously received 5-FU and irinotecan. First line regimens were most commonly based on FOLFOX, FOLFIRI or CAPOX. As patients progressed to later lines of therapy, a greater variability in regimens was observed, suggesting that optimal treatment pathways are ill-defined and increasingly complex beyond second-line therapy.

Patients receiving trifluridine/tipiracil in the UK NPP received a median of 2 (range 1–20) cycles of trifluridine/tipiracil (similar to the German CUP [17]), and had a median treatment persistence of 1.8 (95% CI 1.8–2.4) months, which is broadly similar to the RECOURSE trial population (5.7 weeks) and slightly shorter than reported for the Italian single centre CUP (2.8 months) [10, 13]. The primary reason for treatment discontinuation was disease progression (79%), similar to that reported in United States (USA) expanded access programme (EAP) [23], the Italian (single centre) and Latvian compassionate use programmes [13, 16]. Date of death was not available for most patients in the UK NPP precluding analysis of progression-free survival; however, in patients discontinuing due to disease progression, median progression-free duration was 2.8 (95% CI: 2.4–2.9) months. This is broadly consistent with expectations based on the progression-free survival reported in the RECOURSE trial (2.0 months) [10], Cremolini (2.4 months, 95% CI: 2.2–2.6) [18] and Masuishi et al. (2.1 months) [24] publications, though less than the progression-free survival reported by Skuja et al. (5.0, [95% CI: 4.09–5.90] months), though the authors noted the small sample size (*n* = 14) was a limitation [16]. The tolerability profile of trifluridine/tipiracil was consistent with that previously reported; rates of adverse events were broadly consistent with existing literature, with neutropenia occurring in approximately 30% of patients [14, 15, 17, 23, 25].

### Limitations

Enrolment in the NPP required a clinician’s application, which may have introduced selection bias. Furthermore, patients treated as part of the NPP may not be representative of the patient population treated with trifluridine/tipiracil since marketing authorisation was granted and approval was received from NICE. The details of disease progression were only available in patients discontinuing trifluridine/tipiracil for disease progression and details of death were not available for the majority of patients, which precluded evaluation of progression-free survival and overall survival. Quality of life data was not collected during the NPP. Finally, all data were sourced retrospectively from data documented as part of the NPP, and therefore the quality of this analysis was reliant on the completeness and quality of the data recorded.

## Conclusions

The results of this analysis highlight the wide range and complexity of treatment regimens used to treat patients with mCRC in routine UK clinical practice prior to NICE approval of trifluridine/tipiracil. The tolerability profile of trifluridine/tipiracil was consistent with previous studies, with relatively few patients discontinuing treatment due to AEs.

## Supplementary information

**Additional file 1.** **Table S1.** First line regimen in patients enrolled in the United Kingdom named patient programme. **Figure S1.** First- and second- line treatment sequences in patients who did (S1A) and did not (S1B) receive ≥1 dose of trifluridine/tipiracil.

## Data Availability

Patients provided written consent for data collection, sharing and processing by Servier as part of joining the NPP; however, since this consent does not extend to onwards transmission we are therefore unable to provide access to the data.

## Abbreviations

5-FU: 5-fluorouracil
AE: Adverse event
anti-EGFR: anti-epidermal growth factor receptor
anti-VEGF: anti-vascular endothelial growth factor
CAPOX: capecitabine and oxaliplatin
CI: confidence interval
ECOG-PS: Eastern Cooperative Oncology Group performance status
EMA: European Medicines Agency
FOLFIRI: 5-FU, folinic acid and irinotecan
FOLFOX: 5-FU, oxaliplatin and folinic acid
IQR: interquartile range
mCRC: metastatic colorectal cancer
NHS: National Health Service
NICE: National Institute for Health and Care Excellence
NPP: named patient programme
OS: overall survival
SmPC: summary of medicinal product characteristics
UK: United Kingdom
USPI: United States Prescribing Information
