# Supplementary material for: Review of metastatic colorectal cancer treatment pathways and early clinical experience of trifluridine/tipiracil in the UK named patient programme
Source: BMC Cancer. 2020 Feb 3;20:91. doi: 10.1186/s12885-020-6577-1 (PMC6998075; doi:10.1186/s12885-020-6577-1)
Supplement: Supplementary file 1 — Additional file 1. Table S1. First line regimen in patients enrolled in the United Kingdom named patient programme. Figure S1. First- and second- line treatment sequences in patients who did (S1A) and did not (S1B) receive ≥1 dose of trifluridine/tipiracil. [file 12885_2020_6577_MOESM1_ESM.pdf]

Supplementary figure 1: Treatment pathways in patients with mCRC enrolled in the UK Named Patient Programme who did not receive trifluridine/tipiracil

First line (inner circle) and second line (outer circle) treatments in patients who received ≥1 dose of trifluridine/tipiracil; B) First line (inner circle) and second line (outer circle) treatments in patients who did not receive trifluridine/tipiracil.

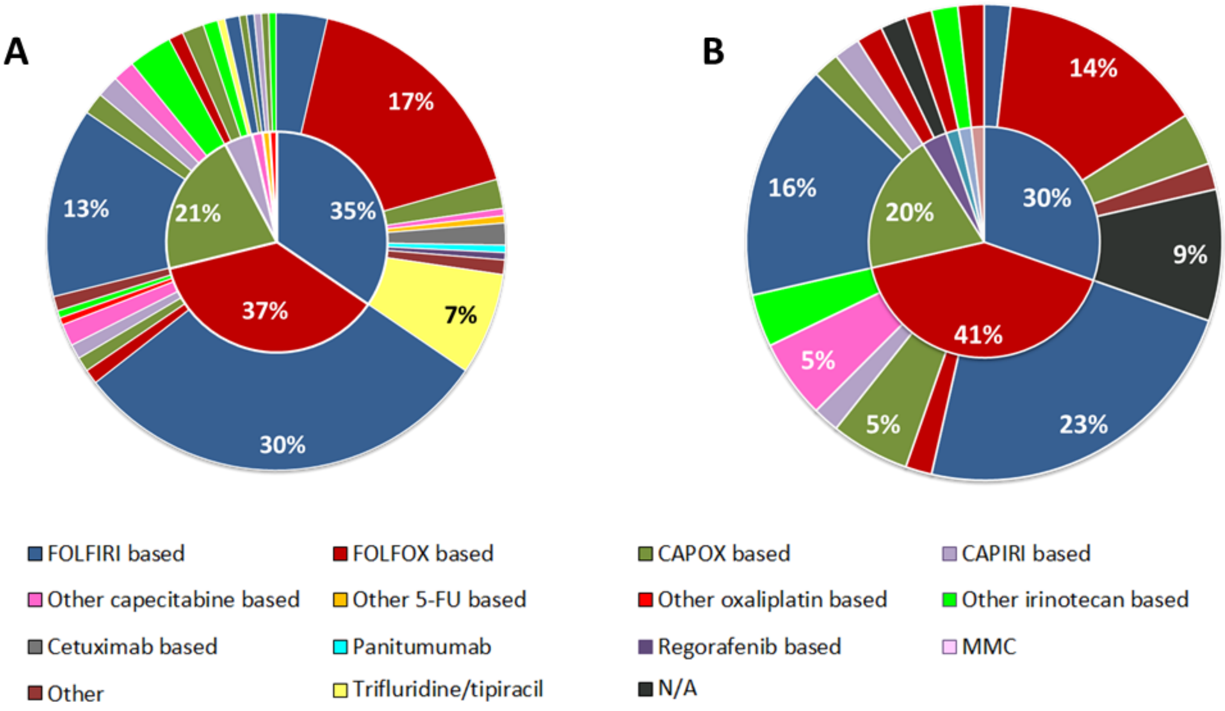

How to interpret: The inner pie chart shows the distribution of first line treatments for mCRC; the outer circle shows the second line treatments that patients in the corresponding segment of the inner circle subsequently received. E.g. chart A shows that 7% of patients received FOLFIRI (first line) followed by trifluridine/tipiracil (second line). Percentages are not presented for groups with <5% of patients. Treatments were grouped based on primary treatment. Treatment regimens were grouped as follows: (i) 'FOLFIRI based' includes FOLFIRI only or in combination with one or more of the following: aflibercept, bevacizumab, capecitabine, cetuximab, panitumumab. (ii) 'FOLFOX based' includes FOLFOX only or in combination with one or more of the following: bevacizumab, capecitabine, cetuximab, irinotecan, other. (iii) 'CAPOX based' includes CAPOX only or CAPOX with bevacizumab and/or other. (iv) 'CAPIRI based' includes CAPIRI only or CAPIRI with bevacizumab or cetuximab. (v) 'Other capecitabine based' includes capecitabine only or in combination with one or more of the following: aflibercept, bevacizumab, mitomycin, other. (vi) 'Other 5-FU based' includes 5-Fluorouracil (5-FU) only or 5-FU plus capecitabine or other. (vii) 'Other oxaliplatin based' includes oxaliplatin only, oxaliplatin with irinotecan and cetuximab and oxaliplatin with other. (viii) 'Other irinotecan based ' includes irinotecan only or in combination with one or more of the following: aflibercept, bevacizumab, cetuximab, other. (ix) 'Cetuximab based' includes cetuximab only or in combination with panitumumab. (x) 'Regorafenib based' includes regorafenib only or with other.

Supplementary table 1: First line regimen in patients enrolled in the United Kingdom Named Patient Programme

First-line regimens for metastatic disease	Patients receiving ≥1 dose of trifluridine/tipiracil	Patients not commencing trifluridine/tipiracil
FOLFIRI	35 (18%)	9 (16%)
FOLFOX	33 (17%)	15 (27%)
CAPOX	29 (15%)	7 (13%)
FOLFOX+BEV	24 (12%)	≤5 (≤9%)
FOLFIRI+CET	16 (8%)	≤5 (≤9%)
FOLFIRI+BEV	14 (7%)	≤5 (≤9%)
CAPOX+BEV	10 (5%)	≤5 (≤9%)
FOLFOX+CET	7 (4%)	≤5 (≤9%)
CAPIRI	≤5 (≤3%)	≤5 (≤9%)
Capecitabine	≤5 (≤3%)	≤5 (≤9%)
CAPIRI+BEV	≤5 (≤3%)	0
FOLFOXIRI+BEV	≤5 (≤3%)	0
FOLFOX+other	≤5 (≤3%)	0
CAPIRI+CET	≤5 (≤3%)	≤5 (≤9%)
5-FU+BEV	≤5 (≤3%)	0
CAPOX+BEV+other	≤5 (≤3%)	0
CAPOX-other	≤5 (≤3%)	≤5 (≤9%)
FOLFIRI+AFL	≤5 (≤3%)	0
FOLFIRI+other	≤5 (≤3%)	≤5 (≤9%)
FOLFOX+BEV+CET	≤5 (≤3%)	0
FOLFOXIRI	0	≤5 (≤9%)
Oxaliplatin	≤5 (≤3%)	0
OXIRI+CET	≤5 (≤3%)	0
FOLFOX+CAP+CET	0	≤5 (≤9%)
Irinotecan	0	≤5 (≤9%)
OX+other	0	≤5 (≤9%)

Groups containing <5 patients have been censored to preserve patient confidentiality.
